# Unraveling the potential of vitamins C and D as adjuvants in depression treatment with escitalopram in an LPS animal model

**DOI:** 10.1007/s10787-023-01404-9

**Published:** 2024-01-05

**Authors:** Omar Gammoh, Rand T. Akasheh, Esam Qnais, Sara Al-Taber, Rabaa Y. Athamneh, Amin A. Hafiz, Abdelrahim Alqudah, Alaa A. A. Aljabali, Murtaza M. Tambuwala

**Affiliations:** 1 Department of Clinical Pharmacy and Pharmacy Practice, Faculty of Pharmacy, Al Yarmouk University, Irbid, Jordan; 2 Department of Nutrition and Dietetics, Faculty of Health Sciences, American University of Madaba, Madaba, Jordan; 3 Division of Cancer Prevention and Control, Department of Internal Medicine, The Ohio State University, Columbus, OH USA; 4 Department of Biology and Biotechnology, Faculty of Science, The Hashemite University, Zarqa, Jordan; 5 Department of Medical Laboratory Sciences, Faculty of Allied Science, Zarqa University, Zarqa, 13133 Jordan; 6 Department of Clinical Nutrition, Faculty of Applied Medical Sciences, Umm AI­Qura University, Mecca, Kingdom of Saudi Arabia; 7 Department of Clinical Pharmacy and Pharmacy Practice, Faculty of Pharmaceutical Sciences, The Hashemite University, Zarqa, Jordan; 8 Department of Pharmaceutics and Pharmaceutical Technology, Faculty of Pharmacy, Yarmouk University, Irbid, 21163 Jordan; 9 Lincoln Medical School, University of Lincoln, Brayford Pool Campus, Lincoln, LN6 7TS UK

**Keywords:** Vitamins C and D, Escitalopram, Depression treatment, Nrf2 pathway, BDNF modulation, Hippocampal response, Chronic LPS model

## Abstract

Depression is linked with oxidative stress and inflammation, where key players include nitric oxide (NO), nuclear factor erythroid 2-related factor 2 (Nrf2), Brain-Derived Neurotrophic Factor (BDNF), and Heme Oxidase-1 (HO-1). Augmenting the efficacy of antidepressants represents a compelling avenue of exploration. We explored the potential of vitamins C and D as adjuncts to escitalopram (Esc) in a lipopolysaccharide (LPS)-induced depression model focusing on the aforementioned biomarkers. Male Swiss albino mice were stratified into distinct groups: control, LPS, LPS + Esc, LPS + Esc + Vit C, LPS + Esc + Vit D, and LPS + Esc + Vit C + Vit D. After a 7-day treatment period, a single LPS dose (2 mg/kg), was administered, followed by comprehensive assessments of behavior and biochemical parameters. Notably, a statistically significant (*p* < 0.05) alleviation of depressive symptoms was discerned in the Esc + Vit C + Vit D group versus the LPS group, albeit with concomitant pronounced sedation evident in all LPS-treated groups (*p* < 0.05). Within the cortex, LPS reduced (*p* < 0.05) the expression levels of NO_*x*_, Nrf2, BDNF, and HO-1, with only HO-1 being reinstated to baseline in the LPS + Esc + Vit D and the LPS + Esc + Vit C + Vit D groups. Conversely, the hippocampal NO_*x*_, Nrf2, and HO-1 levels remained unaltered following LPS administration. Notably, the combination of Esc, Vit C, and Vit D effectively restored hippocampal BDNF levels, which had been diminished by Esc alone. In conclusion, vitamins C and D enhance the therapeutic effects of escitalopram through a mechanism independent of Nrf2. These findings underscore the imperative need for in-depth investigations.

## Introduction

Depression stands as a prevalent global mental health concern, bearing substantial disability implications (Mathers 2008). Its pathophysiology is intricate and multifaceted, encompassing various hypotheses that encompass deficiencies in monoamines, oxidative stress, and inflammatory processes (Köhler et al. 2016; Sowa-ku et al. 2018).

Nitric Oxide (NO_*x*_) assumes a pivotal role in the context of depression, operating as a mediator in oxidative stress and inflammation (Gammoh et al. 2017; Jua et al. 2013). Extensive evidence underscores NO_*x*_’s involvement in triggering the activation of nuclear factor erythroid 2-related factor 2 (Nrf2), a prominent regulator implicated in the realms of oxidative stress and inflammation (Um et al. 2011). Nrf2, functioning as a transcription factor, exerts its influence by mitigating both inflammation and oxidative stress, orchestrating the regulation of an array of target cytoprotective and antioxidant genes. This includes its modulation of Brain-Derived Neurotrophic Factor (BDNF) and Heme Oxidase-1 (HO-1) (Cao et al. 2022; Robledinos-Antón et al. 2019). The potential of Nrf2 activation to ameliorate depression has been observed, exemplified by natural activators like resveratrol and curcumin, which demonstrated the capacity to enhance depression in animal models by modulating the expressions of BDNF and HO-1 (Balogun et al. 2003; Porter and O’Connor 2022). Conversely, instances of LPS-induced depression were linked to the suppression of Nrf2 (Yao et al. 2016). This divergent impact of Nrf2 on depression underscores its intricate involvement and emphasizes the nuanced interplay between molecular mechanisms and depressive states. According to the literature, the expression NO, Nrf2, BDNF, HO-1 and other inflammatory players is altered in relation to depressive behavior in specific brain regions namely the cortex and the hippocampus (Rana et al. 2021; Zuo et al. 2022).

Significantly, the antidepressant effects of various medications can be partially attributed to their capacity to diminish NO_*x*_ levels (Dhir and Kulkarni 2007; Jesse et al. 2008). This is further substantiated by the noteworthy observation that l-arginine, serving as a precursor to NO_*x*_, attenuates the antidepressant efficacy of the tricyclic antidepressant imipramine (Harkin et al. 1999). The elevated presence of NO_*x*_ within the context of depression not only leads to a reduction in Nrf2 levels but also hampers the signaling of BDNF. In a contrasting vein, antidepressants not only exert suppression on NO_*x*_ synthesis but also bring about an elevation in Nrf2 levels, ultimately fostering improvements in BDNF signaling and elevating the levels of HO-1 (Hashimoto 2018; Sani et al. 2023).

Clinically, despite their primary status in depression treatment, their lower side effect profile, lower drug and food interactions, selective serotonin reuptake inhibitors (SSRIs), including escitalopram.

According to robust data, the superiority of escitalopram has been confirmed versus other SSRIs and serotonin-norepinephrine reuptake inhibitors (SNRIs) (Kennedy et al. 2009). However, all antidepressants exhibit a delayed onset of action and variable efficacy. The literature underscores that up to 40% of patients may display non-responsiveness to SSRIs (Gammoh and Bashatwah 2023).

The antioxidant and anti-inflammatory attributes of vitamins C and D have been extensively documented (Cruciani et al. 2019; Holford et al. 2020; Molina et al. 2014). However, emerging evidence indicates their potential antidepressive role. Building upon our prior research, mice were treated with vitamins C and D for 7 days, on the 8th day, mice were subjected to acute restrain for 8 h followed by forced swim test and tail suspension tests. The vitamins demonstrated a reduction in the immobility time for both the forced swim and the tail suspension tests, the most commonly used tests for depression. Notably, this effect was associated with the normalization of circulating NO_*x*_ levels (Gammoh et al. 2023). In addition, several studies assessed the efficacy of an SSRI in combination with either Vit C or Vit D with conflicting data (Amr et al. 2013; Sabir et al. 2018; Sahraian et al. 2015).

Another investigation unveiled the potential of vitamins C and D to activate Nrf2/HO-1 pathways (Nakai et al. 2014; Xu et al. 2020), albeit necessitating further substantiation in an inflammatory model of depression. Lipopolysaccharide (LPS), an endotoxin, is injected intraperitoneally to induce neuroinflammation and depressive symptoms in rodents and is a widely used model to investigate depression and inflammation (Kinra et al. 2022; Maes et al. 2012).

The potential augmented antidepressant effects of combining vitamins C and D with escitalopram remain unexplored within the existing literature.

Therefore, the present study aimed to examine the potential augmented antidepressant effects of combining vitamins C and D with escitalopram and to encompass a comprehensive scrutiny of NO_*x*_, Nrf2, BDNF, and HO-1 expressions in both the hippocampus and frontal cortex in an LPS-induced depression mice model.

## Materials and methods

### Animals

Male Swiss albino mice, acquired from the Animal House Facility of The Hashemite University in Jordan, constituted the subjects for this study. The mice, spanning an age range of 6–8 weeks and exhibiting a weight range of 25–30 g, were enlisted for experimentation. These rodents were housed within distinct cages, maintaining a consistent temperature of 25 °C, along with humidity levels oscillating between 50 and 60%, coupled with ongoing air ventilation. Mice were exposed to 12 h light/12 h dark cycle, all behavioral tests were carried out between 10:00 and 15:00. Ethical considerations were meticulously adhered to throughout this research, aligning with the globally accepted ethical norms governing the treatment and utilization of laboratory animals. The study’s conduct was sanctioned by both the Yarmouk University Institutional Review Board and the Deanship of Scientific Research, with the project being formally registered under the designation of Project Number (51/2022).

### Study design and antidepressant treatments

After a 3-day acclimatization period in the laboratory, the mice were subjected to a random allocation process, leading to their assignment into distinct groups (*n* = 4–6 per group). These categorized groups encompassed: control (saline), Lipopolysaccharide (LPS 2 mg/kg), LPS + escitalopram (Esc 10 mg/kg/day), LPS + Esc + Vitamin C (10 mg/kg/day), LPS + Esc + Vitamin D (1200 IU/kg/week divided into 3 doses), and LPS + Esc + Vitamin C + Vitamin D, treatments were administered for 7 days using the oral route by gavage. The choice of escitalopram was based on its superiority over other antidepressants (Kennedy et al. 2009). The chosen dosages and duration were based on previous work (Gammoh et al. 2023). On the culmination of the treatment regimen, specifically on the 7th day, mice within all groups—except for the control group—received a single intraperitoneal dose of LPS (2 mg/kg) that was freshly prepared using saline. This design was employed since the study aimed to prevent the maladaptive behavior that arises due to a single high dose of LPS to mimic an early inflammatory depression model mirroring a methodology previously detailed in Kinra et al. (2022). Following this, behavioral assessments were executed precisely 2 h after the LPS administration (intraperitoneally), namely the open field test followed by the forced swim test. Afterward, mice were sacrificed.

These dosages of escitalopram, vitamins C and D were chosen from literature and our previous work (Gammoh et al. 2023). Escitalopram pure powder was supplied as a general gift from Pharma International Company, Vitamin C tablets were purchased from Bayer, Vitamin D was purchased from MS Pharma, and LPS was purchased from Santa Cruz.

### Behavioral paradigms

#### Forced Swim Test (FST)

The Forced Swim Test (FST) stands as a widely employed behavioral paradigm for assessing antidepressant-like activity in rodents, where an elevation in the duration of immobility mirrors an augmented state of depression (Porsolt et al. 1979). In essence, individual mice were situated within an unobstructed glass chamber (25 × 15 × 25 cm^3^), containing pristine water maintained at a consistent temperature of 26 ± 1 °C. The entire duration of this test spanned 5 min. The term “Immobility time” (IT) was designated to signify the interval during which mice exhibited complete immobility while submerged in the water. Consequently, an elongated period of floating time emerged as an indicator of heightened depressive behavior. This parameter functioned as the basis for evaluating the antidepressant-like potential of the experimental compounds.

#### Open Field Test (OFT)

The Open Field Test (OFT) was executed to evaluate both locomotion and sedation tendencies in the mice under study (Dishman et al. 1988). Succinctly, the mice were positioned within a central square of the field with dimensions of (72 cm × 72 cm) and granted unrestricted movement for 5 minutes.

The OFT consists of equally divided squares, the number of lines crossed represents the locomotion activity, anxiety, and sedation. While the rearing frequency reflects the sedation and anxiety. The designated testing arena was housed within a test room, illuminated by indirect lighting, and steps were taken to minimize extraneous disturbances and noise. Notably, a meticulous cleaning procedure involving 70% ethyl alcohol was implemented to sterilize the open field maze between each mouse’s trial. The ensuing data encompassed the quantification of locomotion activity, epitomized by the number of lines crossed, and the assessment of sedation, delineated by the rearing frequency. All animal behaviors were video recorded and analyzed by a blinded researcher.

### Biochemical tests

Upon the completion of the experimental protocol, the process of tissue collection, dissection, and preparation ensued. Following euthanizing, the hippocampus and cortex were meticulously dissected, and homogenized, and protein extraction was performed using bicinchoninic acid assay (BCA). The expressions of Nrf2, BDNF, and HO-1 within these anatomical regions were quantified through the utilization of ELISA kits, procured from Sunlong Biotech in China, adhering to the stipulated guidelines provided by the manufacturer. To prepare the tissue samples for ELISA analysis, a rigorous homogenization, and RIPA lysis buffer and procedure were implemented. Tissue samples were homogenized in an appropriate buffer solution, ensuring a thorough disruption of cellular membranes and facilitating the extraction of target proteins. After homogenization, the lysates were then subjected to centrifugation to eliminate debris and cellular particulates, thereby yielding a clarified supernatant containing the target proteins of interest. For quantification, the protein concentration in the clarified lysates was determined using established assays, such as the BCA assay. This concentration data was subsequently employed to normalize the measured levels of Nrf2, BDNF, and HO-1 to the total protein content within each sample. The NO_*x*_ levels were measured using a commercially available kit (Sunlong Biotech China) based on Griess reaction according to the manufacturer’s instructions.

### Statistical analysis

Behavioral and biochemical data underwent thorough analysis through a one-way ANOVA, subsequently followed by Tukey’s post hoc examination. The threshold for statistical significance was established at *p* < 0.05. The outcomes are succinctly conveyed as the mean ± standard error of the mean (SEM), ensuring both clarity and precision in presenting the findings.

## Results

### Forced Swim Test

The LPS-treated cohort exhibited a markedly elevated immobility time (*p* < 0.01) in stark contrast to the control group. Notably, when juxtaposed with the LPS group, the LPS + Esc group demonstrated a substantial reduction in floating time (*p* < 0.05). Equally noteworthy, the LPS + Esc + Vit C + Vit D treated group showcased a pronounced decrease in floating time (*p* < 0.01) in comparison to the LPS group but not to the LPS + Esc group, further underscoring the potential alleviating impact of the combined treatment strategy.

The number of floating episodes reflecting the frequency of depressive episodes was assessed. In comparison to the LPS-treated group, the LPS + Esc group demonstrated a significantly (*p* < 0.001) lower incidence of depressive episodes. Furthermore, the LPS + Esc + Vit C group displayed a significantly (*p* < 0.001) reduced occurrence of depressive episodes. Similarly, the LPS + Esc + Vit D group exhibited a significantly (*p* < 0.05) decreased number of depressive episodes. Notably, the LPS + Esc + Vit C + Vit D group also revealed a significantly (*p* < 0.001) diminished frequency of depressive episodes compared to the LPS group but not compared to the LPS + Esc group.

**Fig. 1:**
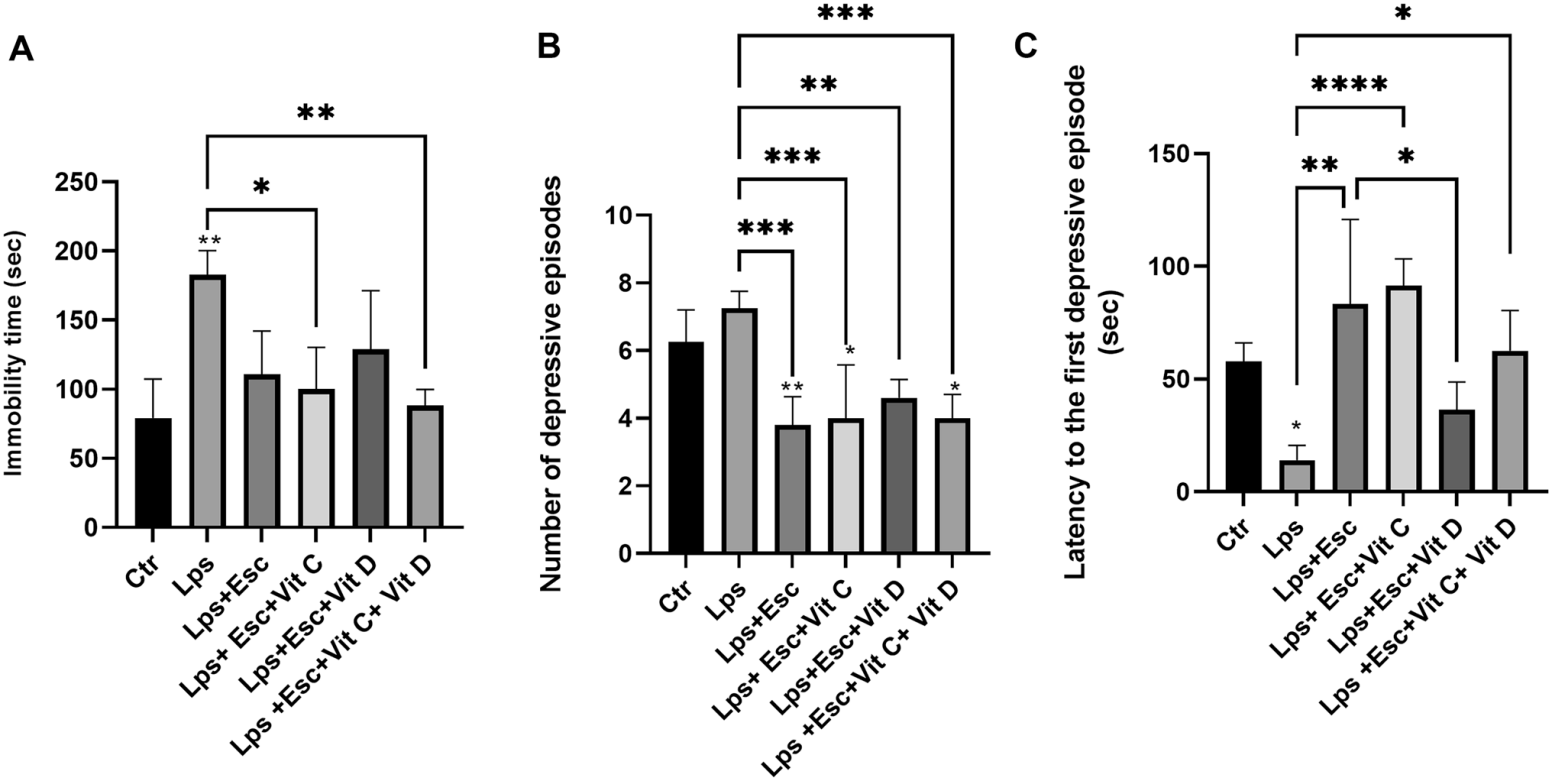
**A** FST floating time among the different groups. ANOVA followed by Tukey’s post hoc analysis. Values are expressed as the mean ± SEM (ANOVA followed by Tukey’s test). *F*(3, 9) = 15.15; *p* = 0.0007, *p < 0.05, **p < 0.01. *Ctr* control, *LPS* lipopolysaccharide, *Esc* escitalopram, *Vit C* vitamin C, *Vit D* vitamin D, *SEM* standard error of the mean. **B** FST number of depressive episodes among the different groups. ANOVA followed by Tukey’s post hoc analysis. Values are expressed as the mean ± SEM (ANOVA followed by Tukey’s test). *F*(5, 22) = 9.91; *p* < 0.0001, *p < 0.05, **p < 0.01, ***p < 0.001. *Ctr* control, *LPS* lipopolysaccharide, *Esc *escitalopram, *Vit C* vitamin C, *Vit D* vitamin D, *SEM* standard error of the mean. **C** FST latency time to the first depressive episode among the different groups. ANOVA followed by Tukey's post hoc analysis. Values are expressed as the mean ± SEM (ANOVA followed by Tukey's test). *F*(5, 18) = 10.78; *p* < 0.0001, *p < 0.05 versus Ctr, ^$^*p* < 0.05 versus LPS, ^#^*p* < 0.05, ^##^*p* < 0.001, ^###^*p* < 0.0001. *Ctr* control, *LPS* lipopolysaccharide, *Esc *escitalopram, *Vit C* vitamin C, *Vit D* vitamin D, *SEM* standard error of the mean

The latency time to the onset of the initial depressive episode was measured. Contrasting the control group, the LPS-treated group exhibited a significantly (*p* < 0.05) shorter latency time. Conversely, the LPS + Esc group demonstrated a notably extended latency time (*p* < 0.001) compared to the LPS group. Similarly, the LPS + Esc + Vit C group displayed a markedly prolonged latency time (*p* < 0.001) relative to the LPS group. Moreover, the LPS + Esc + Vit D group revealed a significantly (*p* < 0.05) increased latency time compared to the LPS group. Impressively, the LPS + Esc + Vit C + Vit D group showed a significantly (*p* < 0.001) higher latency time than the LPS group. Refer to Fig. 1 for a visual representation of the FST results.

### Open field test

**Fig. 2:**
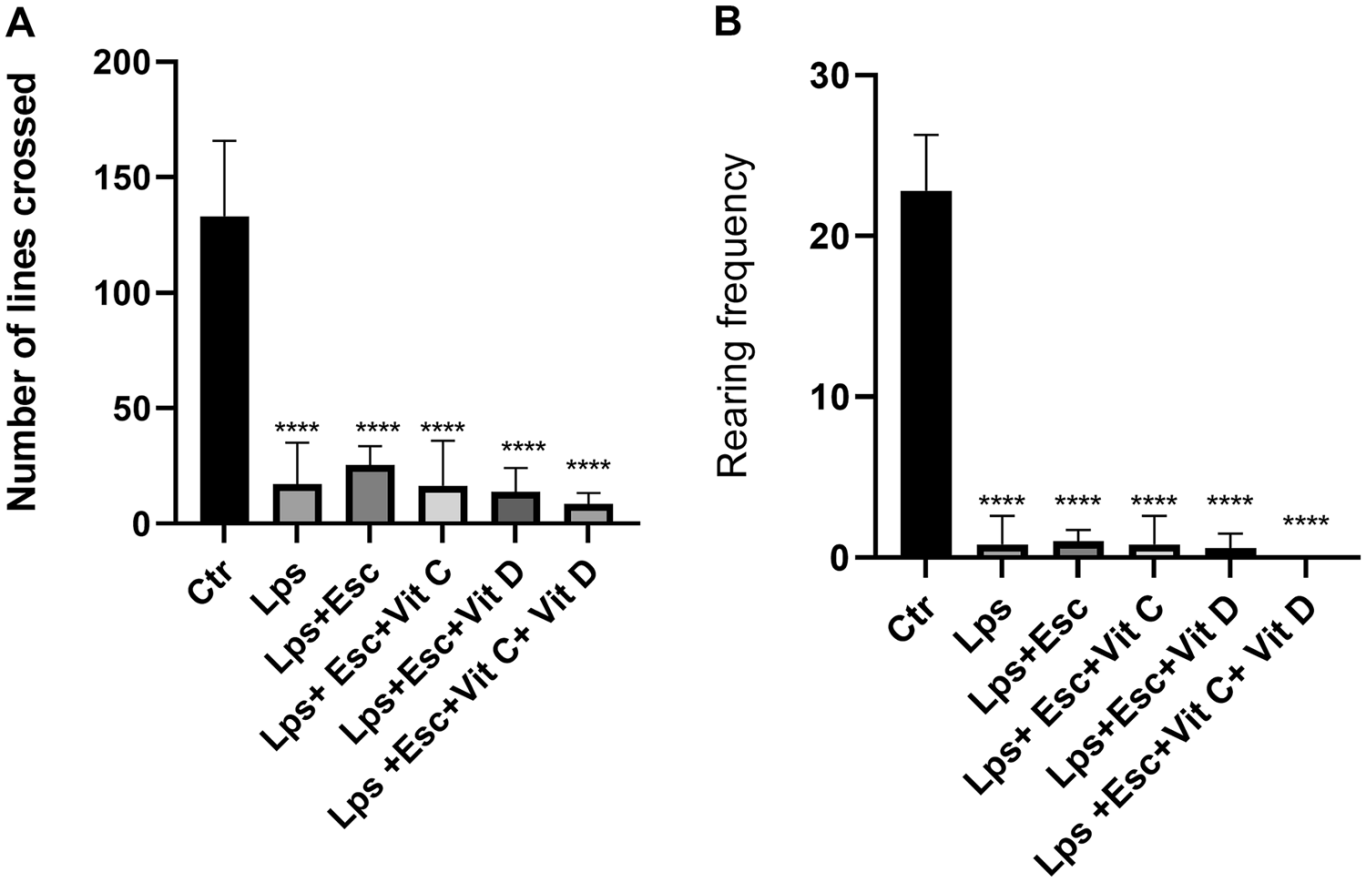
**A** OFT ambulation frequency among the different groups. ANOVA followed by Tukey's post hoc analysis. Values are expressed as the mean ± SEM (ANOVA followed by Tukey's test). *F*(5, 24) = 34.88; *p* < 0.0001, ****p < 0.0001 versus Ctr. *Ctr* control, *LPS* lipopolysaccharide, *Esc *escitalopram, *Vit C* vitamin C, *Vit D* vitamin D, *SEM* standard error of the mean. **B** OFT rearing frequency among the different groups. ANOVA followed by Tukey's post hoc analysis. Values are expressed as the mean ± SEM (ANOVA followed by Tukey's test). *F*(5, 24) = 123.60; *p* < 0.0001, ****p < 0.0001 versus Ctr. *Ctr* control, *LPS* lipopolysaccharide, *Esc *escitalopram, *Vit C* vitamin C, *Vit D* vitamin D, *SEM* standard error of the mean

The open field test (OFT) was utilized to assess the locomotion activity, anxiety levels, and sedation of the mice. Ambulation frequency (AF) was measured as an indicator of mobility. Comparing the treated groups to the control group, all the treated groups exhibited a remarkable reduction in AF (*p* < 0.0001). Likewise, the rearing frequency, another marker of behavior, displayed a significant decrease in all the treated groups when compared to the control group (*p* < 0.0001). For a visual representation of the OFT outcomes refer to Fig. 2.

### Nrf2 expression

**Fig. 3:**
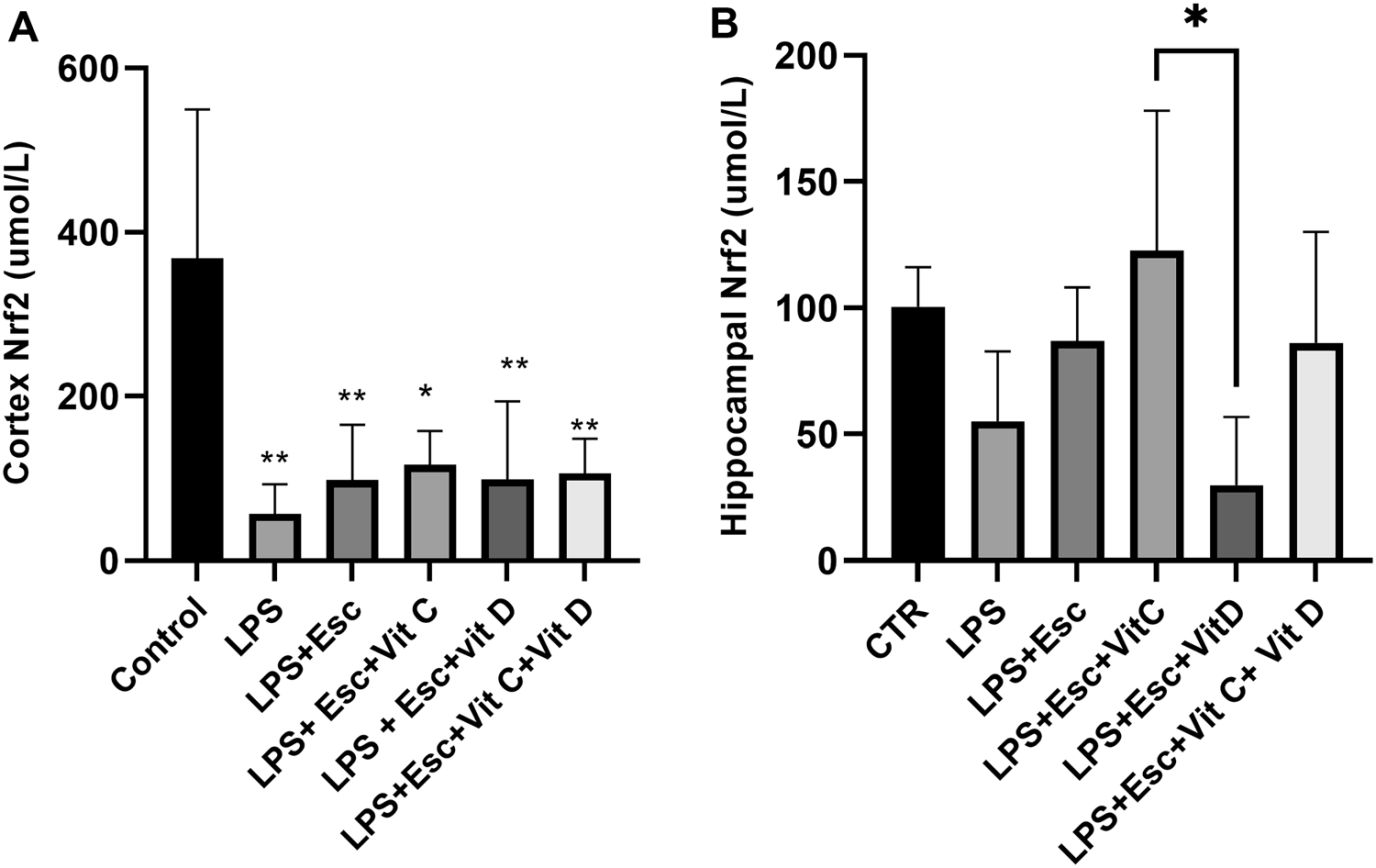
**A** Cortex Nrf2 expression among the different groups. ANOVA followed by Tukey's post hoc analysis. Values are expressed as the mean ± SEM (ANOVA followed by Tukey's test). *F*(5, 19) = 6.23; *p* = 0.001. *p < 0.05, **p < 0.01 vs control, versus Ctr. *Ctr* control, *LPS* lipopolysaccharide, *Esc *escitalopram, *Vit C* vitamin C, *Vit D* vitamin D, *SEM* standard error of the mean. **B** Hippocampal Nrf2 expression among the different groups. ANOVA followed by Tukey's post hoc analysis. Values are expressed as the mean ± SEM (ANOVA followed by Tukey's test). *F*(5, 19) = 3.31; *p* = 0.02. *p < 0.05, versus Ctr. *Ctr* control, *LPS* lipopolysaccharide, *Esc *escitalopram, *Vit C* vitamin C, *Vit D* vitamin D, *SEM* standard error of the mean

Nrf2 expression in both the hippocampus and the cortex was quantified through ELISA analysis. In the cortex, Nrf2 exhibited a significant downregulation (*p* < 0.05) in the LPS-treated group compared to the control group. Similarly, all treated groups displayed a noteworthy downregulation of Nrf2 (*p* < 0.05) when compared to the control group. Conversely, the hippocampal levels of Nrf2 remained relatively unchanged (*p* > 0.05) following LPS insult. It is worth noting that among the treatment groups, only the “LPS + Esc + Vit D” group demonstrated a significantly lower (*p* < 0.05) level of Nrf2 in comparison to the “LPS + Esc + Vit C” group, as depicted in Fig. 3A,B.

### BDNF expression

**Fig. 4:**
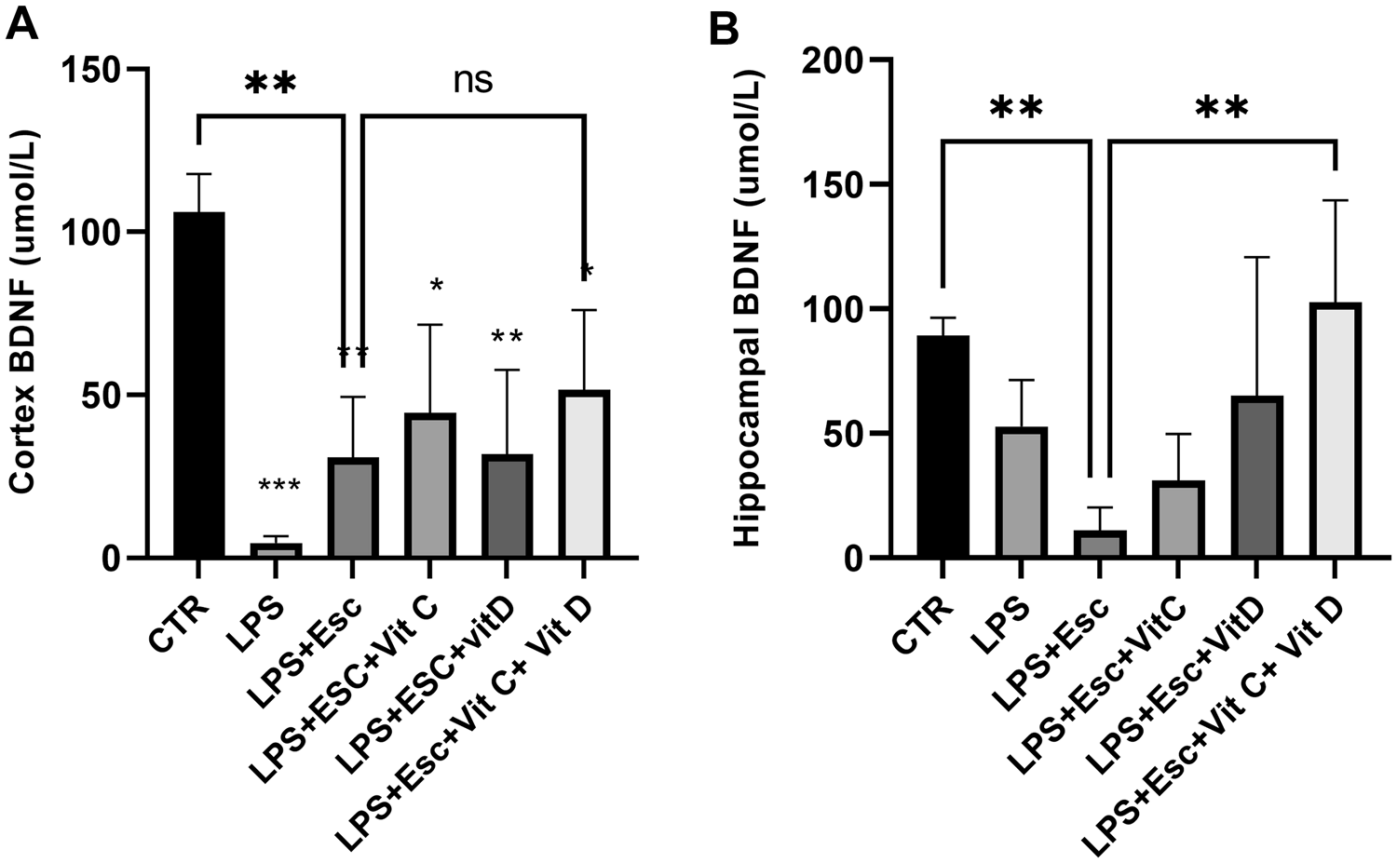
**A** Cortex BDNF expression among the different groups. ANOVA followed by Tukey's post hoc analysis. Values are expressed as the mean ± SEM (ANOVA followed by Tukey's test). *F* (5, 16) = 7.78; *p* < 0.001. *p < 0.05, **p < 0.01, ***p < 0.001 vs control, versus Ctr. *Ctr* control, *LPS* lipopolysaccharide, *Esc *escitalopram, *Vit C* vitamin C, *Vit D* vitamin D, *SEM* standard error of the mean. **B** Hippocampal BDNF expression among the different groups. ANOVA followed by Tukey's post hoc analysis. Values are expressed as the mean ± SEM (ANOVA followed by Tukey's test). *F*(5, 18) = 5.55; *p* = 0.003. **p < 0.001, versus Ctr. *Ctr* control, *LPS* lipopolysaccharide, *Esc *escitalopram, *Vit C* vitamin C, *Vit D* vitamin D, *SEM* standard error of the mean

The quantification of BDNF expression in both the hippocampus and the cortex was carried out using ELISA analysis. In the cortex, a substantial downregulation of BDNF was evident in the LPS-treated group when compared to the control group (*p* < 0.001). Correspondingly, all treated groups displayed a significant reduction in BDNF expression (*p* < 0.05) in comparison to the control group. Conversely, the hippocampal expression of BDNF remained relatively unaffected under the influence of LPS insult (*p* > 0.05). Notably, within the hippocampus, the expression of BDNF was reduced in the LPS + Esc group compared to the control group (*p* < 0.05). In contrast, the combined intervention group exhibited a noteworthy increase in BDNF expression (*p* < 0.05) in comparison to the LPS + Esc group. For a graphical representation of the results, please refer to Fig. 4A,B.

### HO-1 expression

**Fig. 5:**
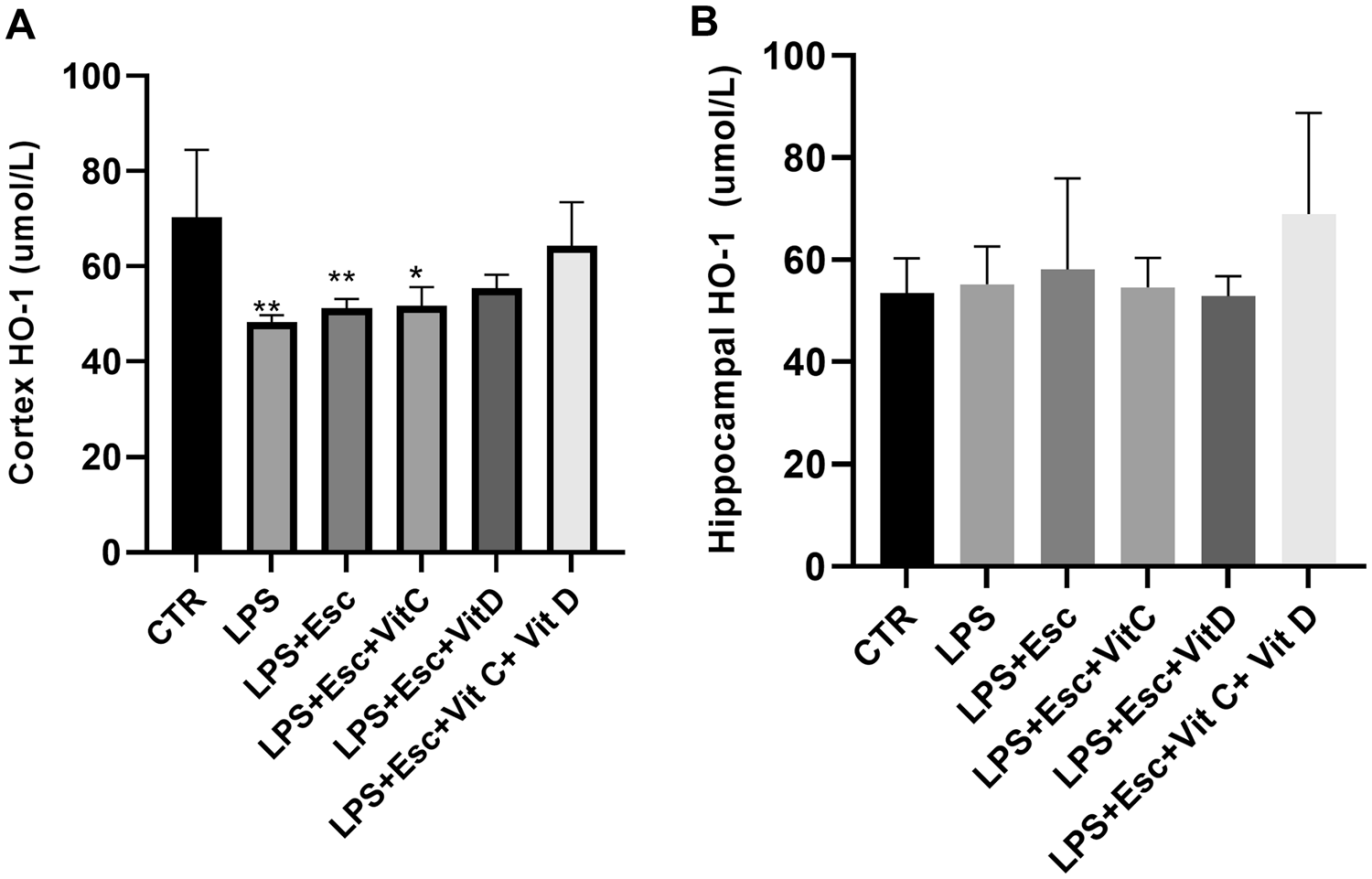
**A** Cortex HO-1 expression among the different groups. ANOVA followed by Tukey's post hoc analysis. Values are expressed as the mean ± SEM (ANOVA followed by Tukey's test). *F*(5, 19) = 5.62; *p* = 0.002. *p < 0.05, **p < 0.01, vs control, versus Ctr. *Ctr* control, *LPS* lipopolysaccharide, *Esc *escitalopram, *Vit C* vitamin C, *Vit D* vitamin D, *SEM* standard error of the mean. **B** Hippocampal HO-1 expression among the different groups. ANOVA followed by Tukey's post hoc analysis. Values are expressed as the mean ± SEM (ANOVA followed by Tukey's test). *F*(5, 21) = 1.13; *p* = 0.38. versus Ctr. *Ctr* control, *LPS* lipopolysaccharide, *Esc *escitalopram, *Vit C* vitamin C, *Vit D* vitamin D, *SEM* standard error of the mean

HO-1 expression was quantified in both the hippocampus and the cortex using ELISA analysis. In the cortex, a significant decrease (*p* < 0.05) in HO-1 expression was observed in the LPS, LPS + Esc, and LPS + Esc + Vit C groups. Intriguingly, the cortical expression of HO-1 did not exhibit a decrease in the combination group. In contrast, the hippocampal HO-1 expression remained consistent across all groups (*p* > 0.05). For a visual representation of these results, please refer to Fig. 5A,B.

### NO_*x*_ levels

The levels of NO_*x*_ were quantified in both the hippocampus and cortex using the Griess reaction kit. Within the cortex, a significant reduction (*p* < 0.05) in NO_*x*_ levels was observed in the LPS, LPS + Esc, LPS + Esc + Vit D, and the combination groups when compared to the control group. Interestingly, the LPS + Esc + Vit C group did not exhibit a decrease in NO_*x*_ levels relative to the control group.

**Fig. 6:**
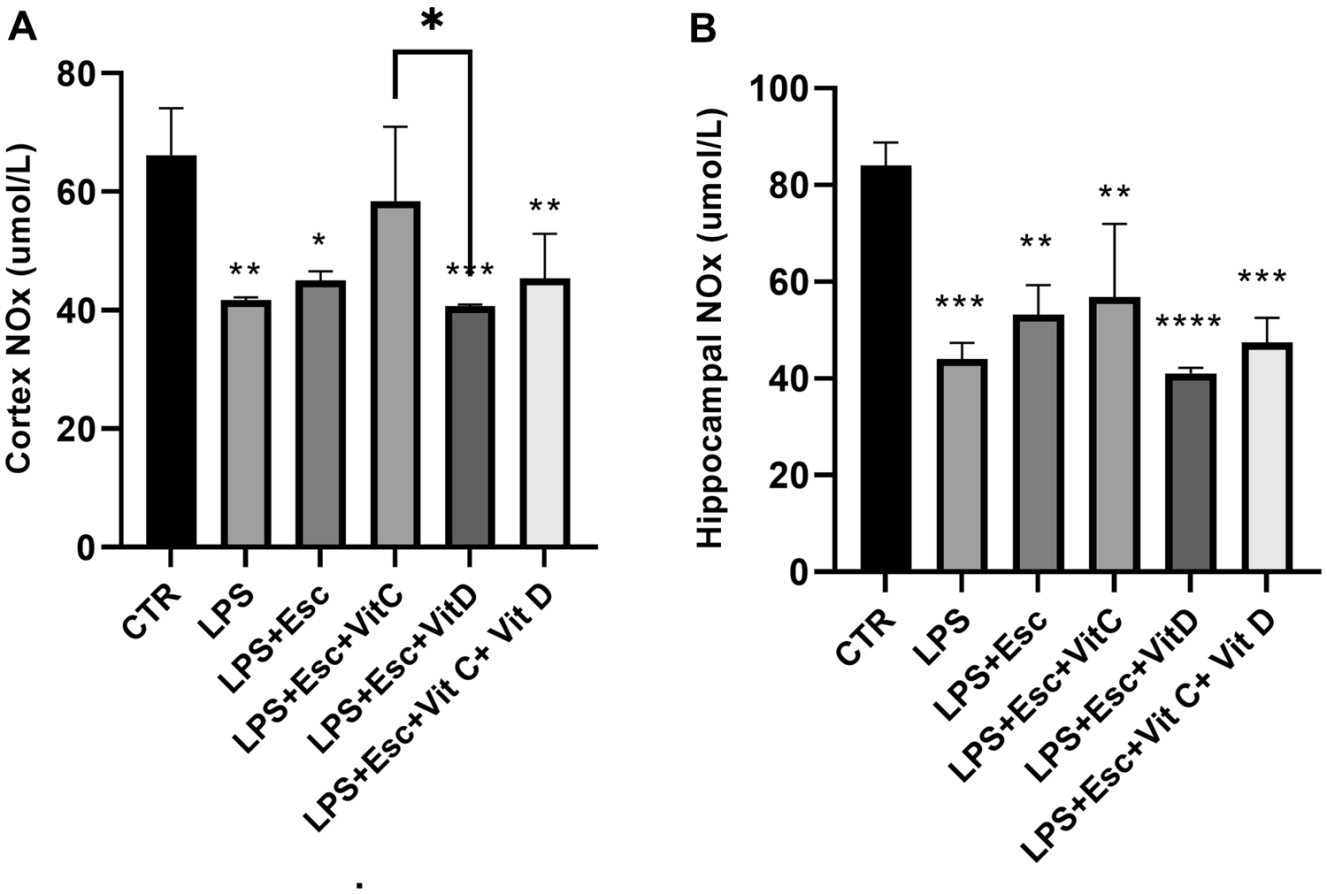
**A** Cortex NO_*x*_ expression among the different groups. ANOVA followed by Tukey's post hoc analysis. Values are expressed as the mean ± SEM (ANOVA followed by Tukey's test). *F*(5, 19) = 8.83; *p* < 0.001. *p < 0.05, **p < 0.01, ***p < 0.001 vs control, versus Ctr. *Ctr* control, *LPS* lipopolysaccharide, *Esc *escitalopram, *Vit C* vitamin C, *Vit D* vitamin D, *SEM* standard error of the mean. **B** Hippocampal NOx expression among the different groups. ANOVA followed by Tukey's post hoc analysis. Values are expressed as the mean ± SEM (ANOVA followed by Tukey's test). *F*(5, 18) = 12.48; *p* < 0.001. **p < 0.01, ***p < 0.001, ****p < 0.0001, versus Ctr. *Ctr* control, *LPS* lipopolysaccharide, *Esc *escitalopram, *Vit C* vitamin C, *Vit D* vitamin D, *SEM* standard error of the mean

Turning our attention to hippocampal NO_*x*_ levels, a notable reduction (*p* < 0.05) was evident in the LPS, LPS + Esc, LPS + Esc + Vit C, LPS + Esc + Vit D, and the combination groups compared to the control group. For a visual representation of these findings, please refer to Fig. 6A,B.

## Discussion

The present work aimed to evaluate the potential antidepressant effects of combining vitamins C and D with escitalopram in relation to the expression of Nrf2, BDNF, HO-1, and NO_*x*_ in the hippocampus and the frontal cortex of LPS-induced model in mice.

Combining Esc with vitamins C and D yielded promising preliminary results. We report that the combination group treated with (LPS + Esc + Vit C + Vit D) reported a superior antidepressant property compared to the (LPS + Esc) group as seen in the floating time. Also, in regards to the number of depressive episodes, the (LPS + Esc + Vit C + Vit D) reported a significant improvement in depressive symptoms versus the LPS group which was similar compared to (LPS + Esc) group. Furthermore, in regard to the latency of depressive symptoms, the (LPS + Esc + Vit C + Vit D) reported a significant improvement versus the LPS group, an effect that was similar compared to (LPS + Esc) group. Also, we report that the LPS dose resulted in severe behavior sickness as seen in the OFT where profound sedation was evident in all the LPS-treated groups.

The present study challenged Swiss/albino male mice after 7 days of the treatments with a single high dose of LPS (Kinra et al. 2022). According to the FST results, escitalopram, when combined with vitamins C and D showed an improvement in the floating time. We report that the combination therapy demonstrated significant improvement versus the LPS group, however, the combination was not superior to Esc alone. This finding was seen in the number of depressive episodes and the latency to the first depressive episode. Our previous results demonstrated the antidepressant roles of vitamins C and D separately in a stress model (Gammoh et al. 2023). This is the first time that these vitamins are challenged in an LPS model. Although the vitamins enhanced the efficacy of Esc, this potentiation was found to be limited. Possible explanations for this modest potentiation could be attributed to the model used and the underlying mechanisms of these vitamins in depression. In our study, Swiss albino male mice were intolerant to the chronic administration of LPS, therefore according to literature a single dose (2 mg/kg) was employed two hours before the behavior tests. This represents an acute inflammatory and behavioral challenge that perhaps did not allow for enough time for the treatments to improve the behavior (Kinra et al. 2022), the second explanation is that vitamins C and D overlapped with the Esc mechanism, in other words, vitamins C and D have serotonergic roles that enhances serotonin brain levels by protecting serotonin from degradation in different brain regions (Gillman 2010; Patrick and Ames 2014), a mechanism that is considered close to the Esc and this explains the lack of synergy of the combination versus Esc alone. Although they could overlap in many mechanisms, however, vitamins C and D have distinct roles, for example vitamin C alone is known to be as a co-factor for dopamine-hydroxylase, therefore enhancing catecholamine synthesis, on the other hand, vitamin D is involved in immune responses, inflammation and neurotrophins synthesis (Shaik-Dasthagirisaheb et al. 2013).

In the cortex, we report that the LPS resulted in a significant decrease in Nrf2, BDNF, HO-1, and NO_*x*_ expression, a decrease that was not normalized by all the treatments administered. Our findings are consistent with literature where LPS was found to diminish Nrf2 in the frontal cortex, subsequently, this leads to a down-regulation of BDNF (Yao et al. 2021). The association between Nrf2 and BDNF has been recently uncovered, upon its activation, Nrf2 upregulates BDNF by interacting at *BDNF* exon I (Cao et al. 2022; Yao et al. 2021). In addition, the relationship between Nrf2 and BDNF was found to be bi-directional, i.e. Nrf2 upregulates BDNF and vice versa (Porter and O’Connor 2022). Moreover, in our study, the Nrf2/HO-1 axis was deactivated under LPS and was not normalized with the various treatments. Our findings are consistent with literature that confirms that HO-1 activation is in part Nrf2-dependent (Paudel et al. 2018; Robledinos-Antón et al. 2019). In addition, our findings suggest a decrease in the NO_*x*_ in the cortex in response to LPS. The implication of NO_*x*_ in depression is well-established, high NO_*x*_ levels are associated with depressive symptoms, and the NO_*x*_ modulators exert antidepressant effects (D et al. 2001; Gammoh and Bashatwah 2023; Lee et al. 2006). According to evidence, NO_*x*_ activates Nrf2 expression and nuclear translocation by directly modulating the Keap-1 cysteine residue (Um et al. 2011). Our previous studies demonstrated an elevation of NO_*x*_ in stress-induced models, however, in the current study depressive symptoms were associated with decreased NO_*x*_ in the cortex. This may be explained by the pan-inflammatory insult caused by the single high dose of LPS and the short time allowed for recovery.

On the other hand, the expression of the target markers was different in the hippocampus. The hippocampal Nrf2 and HO-1 expressions did not vary in the study groups versus the control. The study used an acute LPS insult followed by behavior and scarification after 2 h, perhaps this finding can be explained based on the involvement of a specific brain region to acute or chronic insult, i.e., perhaps Nrf2 and its downstream genes are initially involved in the frontal cortex before the hippocampus.

The (LPS + Esc) group showed a significant decrease in BDNF compared to the control, this decrease was normalized in the presence of vitamins C and D. Although Little is known about the influence of SSRIs or vitamins C and D on BDNF expression, one study showed that the chronic administration of fluoxetine upregulated cortical BDNF in mice (Mendez-David et al. 2015), on the other hand, our finding is consistent with existing literature vitamin C was shown to upregulate BDNF in chronic stress models, although in that study the upregulation did still lower than the control, however, the mechanism needs further elucidation (Rai 2013), some studies pointed out towards involvement of cognitive restoring function, morphological brain changes of vitamin C beside the antioxidant theory (El-Sokkary and Awadalla 2011; Tagliari et al. 2011).

In addition, vitamin D was shown to upregulate hippocampal BDNF levels and improve depressive symptoms (Xu and Liang 2021). The same study highlighted the implication of the hippocampal BDNF in depression symptoms since the administration of a BDNF-binding protein (TrkB-IgG) reversed the antidepressant role of vitamin D. Although the exact mechanism underlying vitamin D levels with BDNF upregulation is unrevealed yet, however, evidence indicates an involvement of vitamin D receptor in the synthesis of neurotrophins including BDNF (Xu and Liang 2021).

In addition, the hippocampal NO_*x*_ expression was decreased in the LPS group and was not normalized with all the treatments administered. This finding is taken together with the hippocampal expression of Nrf2, where Nrf2 followed the same pattern, this supports that Nrf2 activation could be in part NO_*x*_-dependent.

This study contributed to the literature; however, it has some limitations related to the study animal type and design. The study recruited male Swiss albino mice, this strain is well-known for its reliability in depression-related studies, however, the antidepressant response can be also related to the mice strain used which could lead to outcomes difference. Furthermore, due to the high sensitivity of the mice used, the study employed a single dose of LPS to induce depression and inflammation after 7 days of treatment with Esc and vitamins C and D as the mice did not tolerate lower doses for a longer duration. Therefore, future studies could recruit other mice strains to study the effect of the treatment in chronic conditions.

## Conclusion

In conclusion, this study explored the potential antidepressant effects of combining vitamins C and D with escitalopram in a mouse model induced by LPS. The results revealed promising preliminary findings, particularly in terms of improved depressive symptoms and behavioral outcomes when compared to the group treated with escitalopram alone. However, the combination did not surpass the efficacy of escitalopram by itself, suggesting that there may be limitations to the synergistic effects of these compounds.

Furthermore, the study provided insights into the complex molecular mechanisms at play in depression. In the cortex, LPS-induced inflammation led to significant alterations in the expression of Nrf2, BDNF, HO-1, and NO_*x*_, which were not fully normalized by the treatments administered. These findings align with existing literature highlighting the intricate relationship between Nrf2, BDNF, and NO_*x*_ in depression, where their dysregulation may contribute to the condition.

Interestingly, the hippocampus exhibited distinct responses, with Nrf2 and HO-1 expressions remaining relatively stable across study groups. This regional disparity may suggest that specific brain regions respond differently to acute insults like LPS, with the frontal cortex potentially being more involved in the initial response. The study also demonstrated that the combination of vitamins C and D was able to normalize the decrease in BDNF expression observed in the escitalopram-only group, highlighting a potential role for these vitamins in modulating BDNF levels in the hippocampus.

Despite its contributions, this study has limitations related to the choice of mouse strain and experimental design. Using Swiss albino male mice, while reliable for depression-related research, may introduce strain-specific factors that influence treatment outcomes. Additionally, the use of a single high dose of LPS for a short duration presented challenges in replicating chronic depression conditions. Future research could consider alternative mouse strains and explore the effects of treatment under chronic inflammatory conditions to further elucidate the potential of combining vitamins C and D with escitalopram as an antidepressant therapy. Overall, this study adds valuable insights to the field of depression research and paves the way for further investigations into the synergistic effects of these compounds in different models and conditions.

## Data Availability

All data is securely stored by the corresponding authors and will be made available upon request.
